# Supplementary material for: Anxiety and depression symptoms among a sample of Khartoum civilians during the 2023 Sudan armed conflict: A cross-sectional study
Source: PLoS One. 2024 Jul 25;19(7):e0307648. doi: 10.1371/journal.pone.0307648 (PMC11271933; doi:10.1371/journal.pone.0307648)
Supplement: Attachment — Submitted filename: COMMENTS & SUGGESTION New SUDAN.docx [file pone.0307648.s002.docx]

PLOS ONE

**Manuscript Title**- Anxiety and depression among a sample of Khartoum civilians during the 2023 Sudan armed conflict: A cross-sectional study

**Manuscript Number**: PONE-D-23-30028

Dear Researcher! Thank you for conducting this study. Your manuscript has no line numbers to comment on by line number and page. The method and materials section of this manuscript was not clearly written and understandable.

**Abstract: -** It contains all the scientific content and is written well.

**Introduction** correct as “Background”

**Background:** It is more focused on the history of Sudanese politics and war history than common mental disorders during and after military conflicts, and it is better to focus on and rewrite more facts and gaps about common mental illnesses that need more research and more studies on anxiety and depression disorders among people living in conflict or war zones (Khartoum State).

**Methods**: Materials and methods can be correct as "Methods and materials."

For this cross-sectional study, study setting, design, period, source of population, sample size determination and sampling technique, data collection tools and procedures, operational definition, and data processing and analysis were not separately and clearly written. So, it is better if all the above subtopics under Methods and Materials are rewritten in a brief and clear manner.

It is not clear how study participants were selected from the study population. Did you use probability or non-probability techniques? The authors were expected to state the procedures they had used to select quantitative study participants.

You have stated that “this prospective cross-sectional study was conducted between May 27 and June 19, 2023, during the armed military conflict in Khartoum State, the capital of Sudan.” A prospective study design is quite different from a cross-sectional study. The authors should explain what a prospective cross-sectional study means.

As exclusion criteria, the authors stated that "substance abuse and psychiatric history”. How did you differentiate or screen those individuals who had been abusing substances and living with a psychiatric history? Or, what kind of procedures had been used to screen those people who had been abused by substances and had psychiatric disorders?

You stated that “data were collected using an online self-administered questionnaire through Google Forms due to their publicity." How were study participants selected? What kind of sampling technique had been used?

Data were collected using an online self-administered questionnaire through Google Forms. However, you stated that “we asked respondents" or asking respondents—it seems like a face-to-face interview. Please try to rewrite clearly.

For this study, the English version of the questionnaire was translated to the Arabic version, but its contents were not well addressed.

The Statistical analysis plan can be corrected to “Statistical analysis.”

It is not clear how the data were cleaned, coded, and entered into an Excel sheet.

**Results**: - Demographic Characteristics of Study Participants correct it as” Demographic characteristics of study participants”
 A total of 393 participants were included in this study. However, within the Methods and Materials section, the sample size was 384; these two figures are a little different from each other. The total number of single study participants were 314 (80%), which implies that being single is a risk factor for developing common mental illnesses (anxiety and depression). So why were the majority of your study participants single?

Table 1, Standard age classification was not done

**Residence -** Khartoum State was classified into three sub-cities: Khartoum, Khartounbahri, and Omdurman. What is the significance of classifying Khartoum state into sub-cities for this study?

Marital status – total number of divorced = 6(1.5%)

-total number of widowed 1(0.3)

I think model fitness was not checked for these two variables (divorced and widowed), so try to run the row data again to check its model fitness and merge those less than 5%.

**Prevalence of Anxiety and Depression**

Among study participants, 31% of them exhibit severe anxiety, 28% of them have moderate-to-severe anxiety, and using the cutoff point, 57.3% of study participants were anxiety patients. Similarly, 73% of study participants had depression. What about those study participants who had both anxiety and depression (comorbidity between anxiety and depression)? OR, the prevalence of those studies participants who had met criteria for both anxiety and depression during study period? Similarly, what about other common anxiety disorders, such as PTSD and panic disorder, which are common during and after military conflict?

What is the association between anxiety and depression, according to this study?

I have a concern regarding your study title “Anxiety and depression among a sample of Khartoum civilians during the 2023 Sudan armed conflict: A cross-sectional study”. Because normal anxiety and depressive symptoms are common for people living in war zones, Normal anxiety symptoms are quite different from those of pathological anxiety disorder in the following parameters:

**Intensity: -**in cases of pathological anxiety the person experiences relatively high and/or out of proportion to the situation or circumstances, and in cases of normal anxiety relatively low and/or proportionate to the situation or circumstances.

**Duration**:- In cases of pathological anxiety, the person generally experiences longer-lasting or recurrent symptoms. But, in the case of normal anxiety, it is generally shorter-lasting.

**Preoccupation**: - with anxiety is common among patients with pathological anxiety and not common among those with normal anxiety

**Effects on behavior and functioning**: - Pathological anxiety causes long-standing changes in behavior and impairs functioning. But, generally, it does not affect behavior more than temporarily and does not impair functioning.

**Quality of the experience**: - Pathological anxiety is distressing, overwhelming, and incapacitating. However, normal anxiety is unpleasant, but not too distressing or distressing for a long time. So it is too difficult to differentiate normal anxiety from pathological anxiety by analyzing the collected online data.

It is better to modify your study title to “Anxiety and depression symptoms among a sample of Khartoum civilians during the 2023 Sudan armed conflict: A cross-sectional study” . Because of the above factors, your study results were highly exaggerated. “Among the study participants, 70% had depression, and 57.3% suffered from anxiety."

**Factors Associated with Anxiety and Depression, can be corrected as “Factors associated with anxiety and depression”**

Being married was associated with anxiety. (Being married was associated with twice the risk of anxiety compared to being single (OR = 2.21, 95% CI = 1.11, 4.57, p-value = 0.028). But most literature reports that being married is a protective factor for mental illnesses, including anxiety. The authors expected to justify with scientific evidence why being married is associated with anxiety.

Table 3 and table 4 no standard age classification is done

**Discussion**

**You stated that your study aims** “to document the mental status of residents of Khartoum State during the armed conflict that erupted between the Sudanese army and paramilitary forces on April 15, 2023”. But the mental status of an individual is different from mental illness (mental disorder) in many ways. The mental status of a person is a one-time clinical examination finding that is not similar to or equal to a mental disorder. Mental status clinical findings might change from time to time; however, mental disorders (anxiety and depression) are syndromes that can be characterized by behavioral, emotional and autonomic symptoms and sustain for a long period of time. For instance, general anxiety disorder is not diagnosed based on a month's duration of symptoms; at least six consecutive months of experiencing anxiety symptoms by the person are mandatory.

Your discussion is too shallow to address the discrepancies between previous study findings and your study findings.

You have stated that “Sudanese have witnessed the consequences of military conflict in Libya, Syria, and Yemen. This drove them to lead a non-violent revolution in 2019 as they aimed to have democracy”. Sudanese were not physically witnessing the consequences of conflict, similar to other nations; instead, they had heard and watched the conflict through different media. So what distinguishes Sudanese people from other neighboring countries? According to this study, the exaggerated prevalence of anxiety (57.3%) and depression (73%) was not well justified with scientifically sound

**Strengths and limitations**

Well done, but the main limitation of this study was not stated well.

**Conclusion**

Your recommendations were not in line with your study findings. For example, your study did not assess and identify mental health care gaps, and lack of emergency mental health services in Khartoum State.
